# Ictal lack of binding to brain parenchyma suggests integrity of the blood–brain barrier for ^11^ C-dihydroergotamine during glyceryl trinitrate-induced migraine

**DOI:** 10.1093/brain/aww096

**Published:** 2016-05-27

**Authors:** Christoph J. Schankin, Farooq H. Maniyar, Youngho Seo, Shashidar Kori, Michael Eller, Denise E. Chou, Joseph Blecha, Stephanie T. Murphy, Randall A. Hawkins, Till Sprenger, Henry F. VanBrocklin, Peter J. Goadsby

**Affiliations:** ^1^ 1 Headache Group, Department of Neurology, University of California San Francisco, San Francisco, CA, USA; ^2^ 2 Department of Radiology and Biomedical Imaging, University of California San Francisco, San Francisco, CA, USA; ^3^ 3 Department of Neurology, University Hospital Bern - Inselspital, University of Bern, Bern, Switzerland; ^4^ 4 Headache Group, NIHR-Wellcome Trust, King’s Clinical Research Facility, King’s College London, London, UK; ^5^ 5 MAP Pharmaceuticals, Inc., Mountain View, CA, USA; ^6^ 6 Department of Neurology, Columbia University Medical Center, New York, NY, USA; ^7^ 7 Department of Neurology, DKD Helios Klinik, Wiesbaden, Germany

**Keywords:** migraine, headache, drug treatment, imaging

## Abstract

**
See Dreier (doi: 10.1093/aww112 ) for a scientific commentary on this article.
**

For many decades a breakdown of the blood–brain barrier has been postulated to occur in migraine. Hypothetically this would facilitate access of medications, such as dihydroergotamine or triptans, to the brain despite physical properties otherwise restricting their entry. We studied the permeability of the blood–brain barrier in six migraineurs and six control subjects at rest and during acute glyceryl trinitrate-induced migraine attacks using positron emission tomography with the novel radioligand ^11^ C-dihydroergotamine, which is chemically identical to pharmacologically active dihydroergotamine. The influx rate constant *K _i_* , average dynamic image and time activity curve were assessed using arterial blood sampling and served as measures for receptor binding and thus blood–brain barrier penetration. At rest, there was binding of ^11^ C-dihydroergotamine in the choroid plexus, pituitary gland, and venous sinuses as expected from the pharmacology of dihydroergotamine. However, there was no binding to the brain parenchyma, including the hippocampus, the area with the highest density of the highest-affinity dihydroergotamine receptors, and the raphe nuclei, a postulated brainstem site of action during migraine, suggesting that dihydroergotamine is not able to cross the blood–brain barrier. This binding pattern was identical in migraineurs during glyceryl trinitrate-induced migraine attacks as well as in matched control subjects. We conclude that ^11^ C-dihydroergotamine is unable to cross the blood–brain barrier interictally or ictally demonstrating that the blood–brain barrier remains tight for dihydroergotamine during acute glyceryl trinitrate-induced migraine attacks.

## Teaser

Breakdown of the blood–brain barrier has long been postulated to occur in migraine. Schankin et al. explore this possibility using PET with [ ^11^ C]-dihydroergotamine. The radioligand does not bind to the brain parenchyma at rest or during migraine attacks, suggesting that the blood–brain barrier remains intact during attacks.

## Graphical abstract

**Figure:**
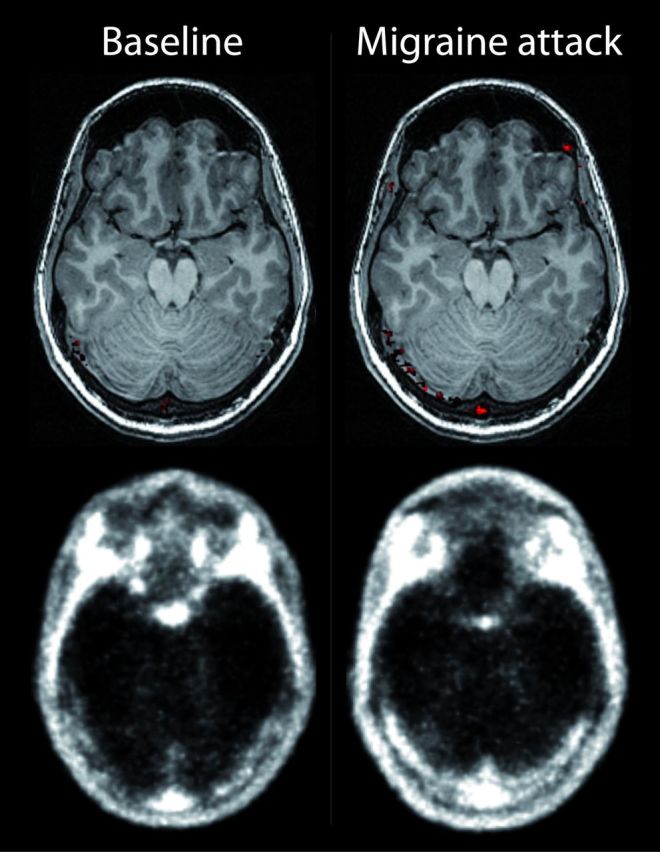
**
See Dreier (doi: 10.1093/aww112 ) for a scientific commentary on this article.
** Breakdown of the blood–brain barrier has long been postulated to occur in migraine. Schankin et al. explore this possibility using PET with [ ^11^ C]-dihydroergotamine. The radioligand does not bind to the brain parenchyma at rest or during migraine attacks, suggesting that the blood–brain barrier remains intact during attacks.

## Introduction

Migraine is a disabling neurological disorder that presents with recurrent attacks of headache ( Goadsby *et al.* , 2002 ), has significant impact on quality of life, and was recently rated number six of the worldwide causes of disability (Global Burden of Disease Study, 2015 #11136). The hypothesis of a blood–brain barrier disruption during acute migraine attacks has been long discussed ( Harper *et al.* , 1977 ), although no human evidence supporting this is available ( Edvinsson and Tfelt-Hansen, 2008 ), save case reports in severe forms of migraine with aura ( Dreier *et al.* , 2005 ; Cha *et al.* , 2007 ). Animal models for migraine aura using cortical spreading depression ( Gursoy-Ozdemir *et al.* , 2004 ) have postulated a mechanism of blood–brain barrier disruption by matrix metalloproteinases, although this too has not been confirmed in humans ( Ashina *et al.* , 2010 ). The question of an ictal blood–brain barrier disruption in migraine is important because whether brain treatments access the brain should drive medicine development, and certainly informs understanding of the site of action of current therapies ( Goadsby, 2013 ).

Here, we tested the hypothesis of blood–brain barrier disruption during migraine attacks in humans, using PET and the effective migraine medication dihydroergotamine (DHE) with *a priori* : (i) low likelihood of interictal blood–brain barrier penetration due to large molecular size ( Ala-Hurula *et al.* , 1979 ); (ii) high sensitivity due to a broad receptor binding profile ( Tfelt-Hansen *et al.* , 2000 ); and (iii) known receptor distribution in humans ( Hall *et al.* , 1997 ).

## Materials and methods

The study was approved by the ethics committee and the radiation safety committee of the University of California, San Francisco (10-05023 and 58605-RU-03-URH), and the US Food and Drug Administration (IND 112,893). All subjects gave written informed consent according to the Declaration of Helsinki. The data have been presented in preliminary form to the 66th Meeting of the American Academy of Neurology ( Schankin *et al.* , 2014 ).

Due to the production of the radioligand solely for the study and arterial blood sampling, exact prediction of the migraine attack was necessary. Therefore, we decided to investigate glyceryl trinitrate (GTN)-triggered migraine attacks, which have been shown to be identical to spontaneous migraine attacks ( Iversen *et al.* , 1989 ).

### Experimental design

**Figure 1:**
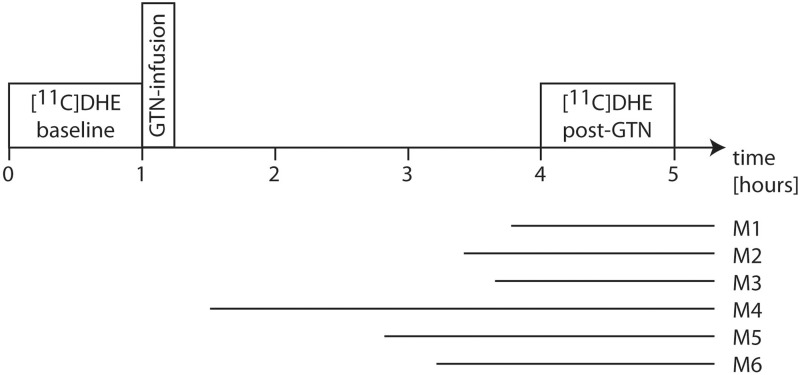
**Study design of the imaging visit** . Every participant had two ^11^ C-DHE PET scans. A baseline pain-free scan was followed by the infusion of GTN, which served as the migraine trigger in migraineurs. The second PET scan was done 3 h after the beginning of the GTN infusion (post-GTN). The horizontal bars illustrate the headache phase of in the migraine patients (Patients M1–6).

Given the excellent binding characteristics of DHE ( Berde and Schild, 1978 ; Goadsby and Gundlach, 1991 ) and our ability to make a high specific activity ligand of not less than 11.7 Ci/nmol measured during quality control, we decided on a qualitative approach to detect a disruption of the blood–brain barrier for DHE in each of six migraineurs and six control subjects. Patients were healthy except for a history of migraine with or without aura. Exclusion criteria were comorbid conditions or the intake of any regular medication. Migraineurs and control subjects were further recruited based on their history of episodic migraine and response to GTN trigger: migraineurs were eligible for the PET scanning part of the study when they developed a migraine attack following GTN ( Headache Classification Committee of the International Headache Society, 2013 ); and control subjects when they remained headache-free. The GTN trigger was done as previously described and validated ( Iversen, 2001 ): subjects received an intravenous infusion of 0.5 µg kg ^−1^ min ^−1^ GTN over 20 min, and clinical characteristics as well as vital signs were recorded during infusion and afterwards for at least 4 h. Of 24 migraineurs recruited, we could not trigger acute migraine attacks in 13 patients, and five withdrew for personal reasons; of 10 control subjects, two reported headache during or after GTN-infusion, one developed significant nausea during the GTN infusion resulting in discontinuation of the study, and one withdrew for personal reasons. Therefore, six migraineurs [two male, four female, mean age ± standard deviation (SD): 33 ± 7 years, two with history of migraine with aura; demographics and medical history shown in Table 1 ] and six control subjects (two male, four female, 32 ± 9 years) underwent a baseline ^11^ C-DHE PET scan, followed by GTN-infusion and a second ^11^ C-DHE PET scan after 3 h ( Fig. 1 ). Each subject also had an interictal high-resolution T _1_ -weighted anatomical MRI (magnetization prepared rapid acquisition gradient sequence) on a General Electric Signa HDxT 3.0 T scanner (GE Healthcare). During the entire second scan, all migraineurs experienced headache fulfilling the criteria for migraine without aura according to the International Classification of Headache Disorders, third edition-beta (ICHD-3beta, Headache Classification Committee of the International Headache Society, 2013 ) ( Table 1 ), and all controls remained pain-free. One patient (Patient M4) went into a migraine attack immediately at the end of the GTN-infusion, i.e. ∼3 h prior to the beginning of the second scan.

**Table 1:** Characteristics of the headaches induced by glyceryl trinitrate in six migraineurs (Patients M1 to M6) During the entire second ^11^ C-DHE scan, all migraineurs had headache attacks fulfilling the criteria for migraine without aura. None of the controls had experienced any headache during the second scan

	Patient's characteristics							Characteristics of headache attack							
	Age	Gender	MA/MO	Duration (years)	Headache frequency (per month)	Acute medication taken	Regular medication	VRS	Laterality	Pulsating	Photophobia and phonophobia	Nausea and/or vomiting	Movement sensitivity	Duration (h)	Entire scan
M1	39	F	MA	22	8	NSAID	None	3	Right	+	Phonophobia	+	+	6	+
M2	27	F	MO	17	8	Paracetamol	None	7	Right	Pressing	+	+	+	5 ^a^	+
M3	45	M	MA	8	8	NSAID	None	4	Bilateral	Pressing	+	+	+	7 ^a^	+
M4	30	M	MO	6	2	NSAR	None	8	Left	+	Photophobia	+	+	19	+
M5	29	F	MO	23	2	NSAR	None	7	Bilateral	+	+	−	+	5 ^a^	+
M6	26	F	MO	11	6	Sumatriptan	None	6	Right	+	+	+	+	5 ^b^	+

MA/MO = previous headache history of migraine with/without aura; VRS = Verbal Rating scale between 0 for no pain, and 10 for maximum pain; + = present; − = not present.

^a^ Went to sleep with headache.

^b^ Took pain medication.

### Tracer synthesis

^11^ C-DHE was synthesized in a three-step approach from commercially available dihydroergotamine mesylate provided by MAP Pharmaceuticals as shown in detail in the Supplementary material and Supplementary Figs 1 and Supplementary Data . In brief: (i) *N-* dealkylative cyanation of DHE using cyanogen bromide (BrCN) resulted in *N* -cyano-dihydroergotamine (DHE-CN); (ii) DHE-CN was reduced to *N* -desmethyl-dihydroergotamine (desmethyl-DHE) that was used as a stock for all preparations; and (iii) Carbon-11 methylation of desmethyl-DHE was performed with ^11^ C methyl iodide ( ^11^ CH _3_ I) on the FX C Pro methyl iodide synthesis unit. ^11^ C-DHE was purified via high-performance liquid chromatography, concentrated on a C18 plus sep-pak and eluted with ethanol into saline. The final solution was sterile filtered and a sample was analysed according to standard procedures.

### Data acquisition

^11^ C-DHE, 10–20 mCi, was injected as an intravenous bolus. Arterial blood samples were taken at the following time points: 1, 2, 3, 4, 5, 10, 15, 30, 45, and 60 min. Dynamic PET scans were acquired over 60 min using a GE Discovery VCT PET/CT scanner (GE Healthcare) in 3D mode with septa retracted. A low-mA CT scan prior to the PET was used for attenuation correction. Images were reconstructed by 3D iterative reconstruction into 47 image planes (separation 3.27 mm) and into a 128 × 128 image matrix (pixel size: 2.1 × 2.1 mm ^2^ ).

### Data analysis

Blood–brain barrier penetration of ^11^ C-DHE was assessed using three complementary approaches:

(i) The influx rate constant *K _i_* (1/min) was used as a measure for the binding of ^11^ C-DHE to the target tissue. *K _i_* was calculated based on the arterial input function using the Patlak graphical analysis implemented in PMOD version 3.307 (PMOD Technologies, Zurich, Switzerland). The Patlak graphical analysis is a linear model of the two-tissue irreversible compartment model, and used to derive *K _i_* on each voxel. This model is appropriate for DHE due to the very long duration of action of DHE *in vitro* ( Müller-Schweinitzer, 1980 ), its low dissociation constant *K _d_* (<60 pM for 5-HT _1B_ receptors), and its slow dissociation (half-life > 2 h) ( Hamblin *et al.* , 1987 ). The average dynamic image was used as reference for coregistration with the structural MRI in SPM8 (Wellcome Department of Imaging Neuroscience, http://www.fil.ion.ucl.ac.uk/spm ) simultaneously coregistering the MRI with the *K _i_* image. The coregistered MRI was segmented into grey matter and white matter using standard parameters. The spatial normalization parameters were applied to spatially normalize the *K _i_* image and the MRI. Areas with *K _i_* > 0.001/min were identified in MRIcron ( http://www.mccauslandcenter.sc.edu/mricro/mricron/index.html ) with the coregistered MRI as background and the *K _i_* map as overlay.
Further, the maximum *K _i_* per volume of interest was chosen as measure for binding of ^11^ C-DHE as it appears less observer-dependent and more reproducible than the mean value ( Adams *et al.* , 2010 ). The maximum *K _i_* was obtained in MRIcron by drawing volumes of interest on the frontal, medial temporal (hippocampus), parietal, and occipital lobe, brainstem (raphe nuclei), as well as thalamus. The volume of interest was spatially normalized as described above.
(ii) The average dynamic image was calculated over the entire scanning period and was compared visually between the post-GTN scan (migraine attack in migraineurs, second scan in controls) and the respective baseline scan.
(iii) Time activity curves were generated in PMOD using volumes of interest over the same regions as in (i). Maximum blood–brain barrier penetration was approximated by comparing the arterial input of the radioligand to the measured mean standardized uptake values (SUV) for each subject, time point (i.e. frame) and volume of interest of the dynamic PET. The effect of migraine attacks in migraineurs and GTN in controls was approximated by comparing the area under the curve using the trapezoid approach.

### Statistics

SPSS (v20, IBM Corp., Armonk, New York, USA) was used for data analysis. Standard descriptive statistics were applied. As the area under the curve of SUV was not normally distributed (assessed with Shapiro-Wilk test) the respective post-GTN state was compared with the baseline state using the Wilcoxon signed-rank test. When appropriate, data are presented as mean ± SD or mean ± standard error of the mean (SEM).

## Results

**Figure 2:**
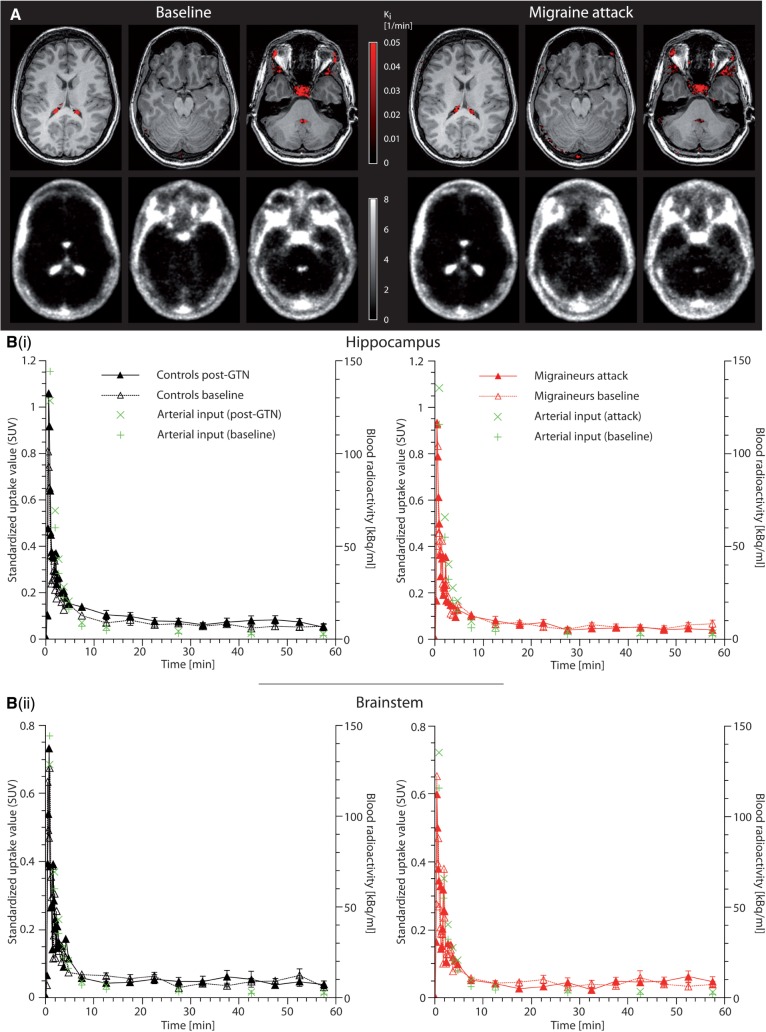
**The blood–brain barrier remains intact during migraine attacks** . ( **A** ) The *K _i_* map in the *upper row* shows the binding pattern of ^11^ C-DHE overlaid on a structural MRI. The average dynamic image is depicted in the *bottom row* . Except for the structures outside the blood–brain barrier, i.e. choroid plexus, pituitary fossa, venous sinus, and facial tissue, there is no intracranial binding of ^11^ C-DHE ( *K _i_* = 0/min). There is only very low average radioactivity in the brain parenchyma. Due to the abundance of DHE receptors in the brain tissue, this indicates that ^11^ C-DHE is not able to cross the blood–brain barrier at baseline state ( *left* ). Notably, there is no change of this pattern during acute migraine attacks ( *right* ) suggesting that the blood–brain barrier remains intact during migraine headache. This was identical for all six migraineurs and control subjects at baseline and in post-GTN state. For illustration purposes, the *K _i_* map of one subject (Patient M6) was plotted at a threshold of *K _i_* > 0.001/min and was overlaid onto the subject’s coregistered MRI. The average dynamic PET was taken from the same subject. ( **B** ) The maximum penetration of the blood–brain barrier was further approximated for migraineurs ( *n = 6* ) and controls ( *n = 6* ) by assessing the time-activity curves (depicting mean for all time points and mean ± SEM between 10 and 60 min for the purpose of clarity) of standardized uptake values (SUV) as exemplarily shown for hippocampus [ **B(i)** ] and brainstem [ **B(ii)** ]. In all areas, the SUV followed the arterial input radioactivity (green crosses). This rapid washout of tracer supports the lack of parenchymal binding of ^11^ C-DHE seen in the *K _i_* maps in **A** . In all parenchymal areas, there was no difference between the area under the curve of the post-GTN (i.e. migraine state in migraineurs) and the respective baseline state (Wilcoxon signed-rank test: *P* > 0.05).

*K _i_* maps in migraineurs and controls demonstrated binding of ^11^ C-DHE ( *K _i_* > 0/min) in the choroid plexus of the lateral and fourth ventricles, the pituitary fossa, venous sinuses, and facial tissue ( Fig. 2 A). There was no binding (maximum *K _i_* = 0/min) in the specific volumes of interest within the frontal, medial temporal (hippocampus), parietal and occipital lobes, the brainstem (raphe nuclei) or the thalamus. The average of dynamic frames (average dynamic image) demonstrated radioactivity in the same intracranial areas ( Fig. 2 A), but none in the brain parenchyma. Similarly, time activity curves in all volumes of interest showed a rapid washout of the radioligand behaving like the arterial input function. Figure 2 B exemplarily illustrates this for the hippocampus and the brainstem (raphe nuclei), which have the highest density of 5HT _1A_ receptors to which DHE binds with high affinity *in vitro* ( Leysen *et al.* , 1996 ; Hall *et al.* , 1997 ).

In the migraine state, there were no differences from baseline with respect to the *K _i_* map and average dynamic image ( Fig. 2 A). The area under the time activity curve did not differ in migraineurs between migraine state and baseline (hippocampus: W = −7; *n*_s/r_ = 6; *P* > 0.05; brainstem: W = 9; *n*_s/r_ = 6; *P* > 0.05; Wilcoxon signed-rank test) or in controls between post-GTN state and baseline (hippocampus: W = 7; *n*_s/r_ = 6; *P* > 0.05; brainstem: W = 4; *n*_s/r_ = 6; *P* > 0.05; Fig. 2 B).

## Discussion

The parenchymal pattern of (i) no binding assessed by calculating the influx rate constant *K _i_* using the gold-standard arterial blood sampling for the input function; (ii) lack of radioactivity in the average dynamic image; and (iii) time activity curves behaving like the arterial input function, indicates that ^11^ C-DHE does not have access to receptors in the brain parenchyma. The main metabolite of ^11^ C-DHE ( Maurer and Frick, 1984 ), ^11^ C-8’-hydroxy-DHE, is also radioactive, and the lack of binding in PET indicates that it does not penetrate the blood–brain barrier either. Delayed binding of DHE or its metabolites to the brain during the first scan preventing binding of ^11^ C-DHE or its metabolites during the second scan after hypothetical blood–brain barrier dysfunction during migraine would explain the lack of binding during migraine. This, however, seems very unlikely due to the low injected mass of DHE for the scans owing to the very high specific activity of our ligand leaving most receptors unbound. The binding pattern further cannot be explained by ^11^ C-DHE being subject to an outside transport by an active efflux system such as P-glycoprotein, as the model P-glycoprotein substrate for PET, ^11^ C-verapamil ( Wagner *et al.* , 2009 ), shows substantially different behaviour when compared to ^11^ C-DHE. Given the identical chemistry and thus pharmacology of DHE and ^11^ C-DHE, the broad receptor profile, including 5HT _1A_ , 5HT _1B_ , 5HT _1D_ receptors, α-adrenoceptors and dopamine D _2_ receptors ( Tfelt-Hansen *et al.* , 2000 ) and the lack of binding in hippocampus and raphe nuclei, areas with the greatest density ( Hall *et al.* , 1997 ) of the high affinity DHE receptors ( Tfelt-Hansen *et al.* , 2000 ), it seems clear the blood–brain barrier is not altered in migraine, at least not for DHE. This is supported by binding to the choroid plexus, which has 5HT _1c_ receptors ( Brown *et al.* , 1991 ) of lower affinity with fenestrated capillaries ( Strazielle and Ghersi-Egea, 2000 ) and probably explains the detection of DHE in the CSF ( Ala-Hurula *et al.* , 1979 ). Similarly, strong binding to the pituitary gland would be expected, given that it is outside of the blood–brain barrier ( Kuhnel, 2003 ) with receptors that would bind DHE ( Lawson and Gala, 1975 ). The absence of binding to the brain parenchyma against this background indicates that there was no exchange from the vasculature to the tissue; in other words, ^11^ C-DHE was not able to cross the blood–brain barrier.

An important limitation of our study is the limited spatial resolution of PET. Due to partial volume effects, areas close to the CSF, such as the periaqueductal grey or the area postrema, cannot be assessed sufficiently ( Soret *et al.* , 2007 ). Thus, the lack of binding in these areas does not answer the question whether DHE, other ergot alkaloids or triptans utilize central mechanisms in areas close to the CSF, without natural blood–brain barrier penetration ( Bartsch *et al.* , 2004 ), or whether they act mainly via peripheral mechanisms ( Saito *et al.* , 1988 ). A possible central mechanism of (other) migraine medication is for instance reflected by the demonstration of the blood–brain barrier passage by some acute migraine medication in healthy volunteers ( Bergstrom *et al.* , 2006 ). However, it needs to be stressed that answering the question of central mechanisms of migraine medication has not been the purpose of our study. A further limitation of the study is that we did not study migraine with aura patients and cannot conclude anything about the state of their blood–brain barrier.

Further, our study results were obtained in GTN-induced migraine with the PET-tracer ^11^ C-DHE, a molecule of 583.7 g/mol. This allows conclusions about blood–brain barrier integrity only for these conditions and not for other acute migraine medications, such as sumatriptan, which has a smaller molecular size (295.4 g/mol). We believe that investigating the ‘per definition’ secondary headache GTN-triggered migraine does not pose a significant bias for several reasons. First, GTN-induced migraine attacks cannot be distinguished from spontaneous migraine attacks on a clinical basis ( Iversen *et al.* , 1989 ) including premonitory symptoms ( Afridi *et al.* , 2004 ). Second, it is typical for migraineurs to experience attacks under certain conditions, i.e. triggers, such as skipping meals or lack of sleep; and thirdly, spontaneous attacks do not differ from GTN-induced migraine on paraclinical studies including the pattern of activation during functional brain imaging ( Afridi *et al.* , 2005 *a* , *b* ). The maximum coverage of migraine attack with this study is ∼4 h in Patient M4 limiting our findings to this period. From a clinical perspective, however, we believe that testing the integrity of the blood–brain barrier for this early phase of the migraine attack is of particular importance, as early treatment when headache is milder, i.e. therapy within 1 h after onset of the attack, has been shown to be significantly more effective than late treatment ( Goadsby *et al.* , 2008 ).

Whether the small amounts of DHE detected in the CSF ( Ala-Hurula *et al.* , 1979 ), or similarly of triptans ( Goadsby, 2000 ; Goadsby and Sprenger, 2010 ) or CGRP receptor antagonists ( Ho *et al.* , 2010 ; Hewitt *et al.* , 2011 ), are sufficient for an effect is unclear. Only blood–brain barrier penetrant medicines will ultimately answer that question. Importantly, the pattern of *K _i_* map, average dynamic image and time activity curve did not change from baseline during acute migraine attacks in all six migraineurs, who had migraine attacks for the duration of the scan. It is unlikely that the application of ^11^ C-DHE has treated the migraine attack successfully resulting in a closure of a (hypothetically) broken-down blood–brain barrier as ^11^ C-DHE was applied at tracer amounts, and patients continued to have migraine attacks over the scan period.

The identical behaviour of ^11^ C-DHE in controls, migraineurs outside and during attacks is the first direct evidence in human that the blood–brain barrier is not altered during migraine without aura, at least not for DHE in GTN-triggered migraines. Therefore, availability of brain sites for certain medicines to access is not altered by migraine attacks. With the limitations mentioned above this changes our understanding of migraine pathophysiology and treatment. The complex pathophysiology of migraine involves increased trigeminovascular activity, which is likely associated with hypothalamic and brainstem dysfunction ( Maniyar *et al.* , 2014 ), such that brain access may be an advantage, for example in preventive treatment approaches. Knowing more about the pathophysiology of migraine makes developing new therapies possible for this most disabling neurological disorder.

## Supplementary Material

Supplementary Data

## Abbreviations

DHE: dihydroergotamine
GTN: glyceryl trinitrate
* K _i_*: influx rate constant
