# Supplementary material for: Ictal lack of binding to brain parenchyma suggests integrity of the blood–brain barrier for 11 C-dihydroergotamine during glyceryl trinitrate-induced migraine
Source: Brain. 2016 May 27;139(7):1994–2001. doi: 10.1093/brain/aww096 (PMC4939703; doi:10.1093/brain/aww096)
Supplement: Supplementary Data [file aww096_supplementary_data.zip › brain-2015-02077-File005.pdf]

Supplementary Text: Tracer synthesis of [^{11}C]-dihydroergotamine.

(i) N-dealkylative cyanation (von Braun reaction): A suspension of 2.5 g dihydroergotamine mesylate (DHE) in 24 mL dry chloroform ($CHCl_3$) was purged with nitrogen gas. 0.75 g cyanogen bromide (BrCN) was added and the reaction was stirred at room temperature for 48 h. The mixture was dried under rotary evaporation and purified by chromatography over silica gel using 100% ethyl acetate to afford 1.07 g (42%) of N-cyano-dihydroergotamine (DHE-CN). (ii) Reduction: 1.07 g of DHE-CN was dissolved in 20 mL of dioxane. Water (2 mL) and a catalytic amount of Raney nickel were added. The reaction was purged with nitrogen gas and a balloon containing H_2 gas was added with a needle that reached the bottom of the stirring solution. The reaction was vented for 10 s to ensure the balloon is forcing H_2 into the solution. The vent needle was removed. The reaction was heated to 50°C for 16 h, filtered to remove the Raney nickel, and dried under rotary evaporation. The resulting N-desmethyl-dihydroergotamine (desmethyl-DHE) was purified by chromatography over silica gel using 5% methanol in dichloromethane resulting in 0.538 g (52%) of a white solid that was used as a stock for all scans. (iii) Hot methylation: Between 0.5 and 1.0 mg of desmethyl-DHE was dissolved in 200 µL of dimethylsulfoxide and added to the reactor on the General Electric FX C Pro methyl iodide box. The [^{11}C]methyl iodide ($^{11}CH_3I$, 500-900 mCi) was bubbled into the precursor solution at 25 °C and the reactor was kept at 100 °C for 2 min. The mixture was diluted with 1 mL of H_2O and injected onto the HPLC. The high-performance liquid chromatography purification was carried out on a phenyl hexyl column (250 x 10 mm, Phenomenex) with a solvent system of 40% acetonitrile/water with 1% trifluoroacetic acid at 7 mL/min. The radioactive peak corresponding to [^{11}C]DHE was collected into the large round bottom flask in the FX C Pro that held 30 ml of water and was passed

through a C18 plus sep-pak. The sep-pak was washed with 3 mL of water and finally eluted with 1 mL of ethanol into the product vial containing 12 mL of saline. The final solution was sterile filtered and a quality control sample (0.5-1.0 mL) was analyzed according to standard procedures at our radiopharmaceutical facility (**Supplementary Fig. 1 and 2**).
