# Supplementary material for: Ictal lack of binding to brain parenchyma suggests integrity of the blood–brain barrier for 11 C-dihydroergotamine during glyceryl trinitrate-induced migraine
Source: Brain. 2016 May 27;139(7):1994–2001. doi: 10.1093/brain/aww096 (PMC4939703; doi:10.1093/brain/aww096)
Supplement: Supplementary Data [file aww096_supplementary_data.zip › brain-2015-02077-File007.pdf]

Supplementary Fig. 1: Synthesis of [^{11}C]-dihydroergotamine.

Commercially available dihydroergotamine was used to synthesize [^{11}C]-dihydroergotamine in a three step procedure using N-dealkylative cyanation (von Braun reaction), reduction, and hot methylation with $^{11}CH_3I$.

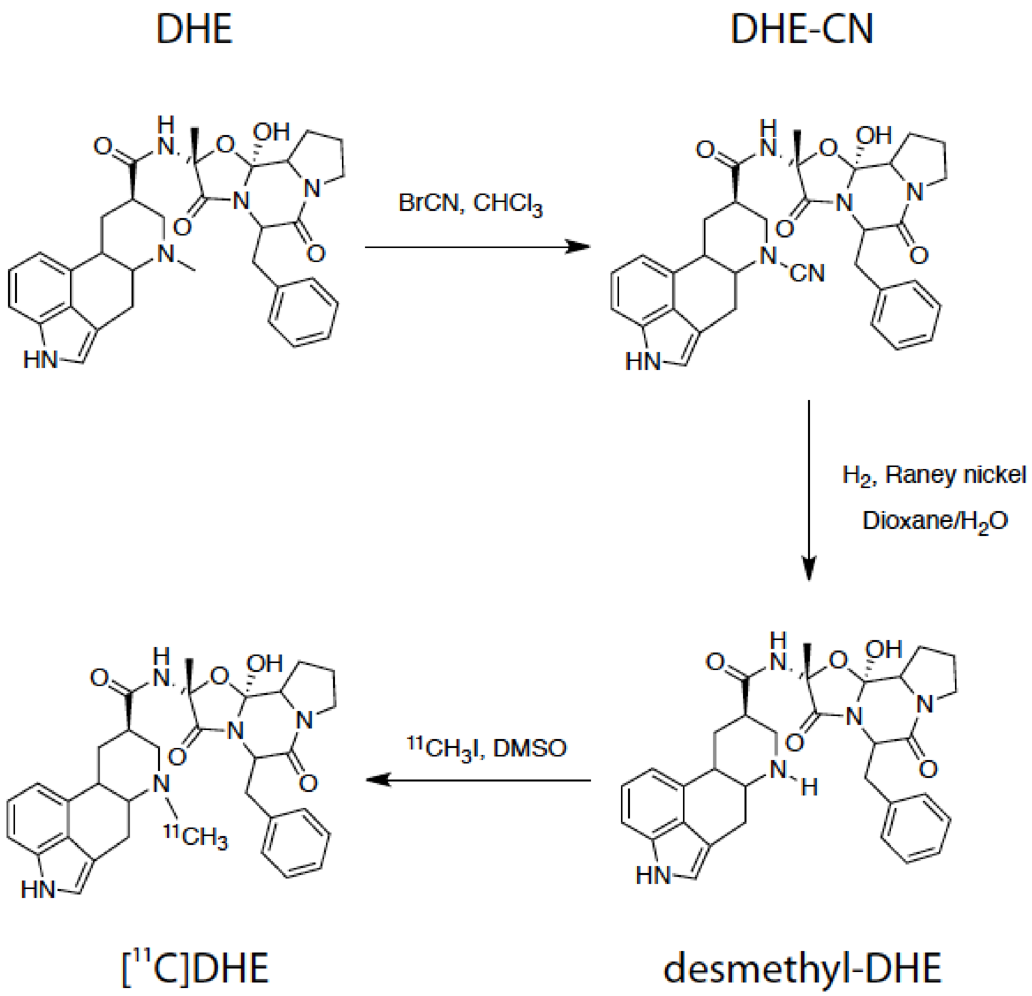
DHE
DHE-CN
BrCN, CHCl3
H2, Raney nickel
Dioxane/H2O
11CH3I, DMSO
[11C]DHE
desmethyl-DHE

Supplementary Fig. 2: [^{11}C]-dihydroergotamine is chemically identical to dihydroergotamine.

High-performance liquid chromatography of [^{11}C]-dihydroergotamine ([^{11}C]DHE) as part of the quality control. Non-radioactive dihydroergotamine (DHE; approximately 5µg) was added to [^{11}C]DHE to ensure identity of the tracer. The outflow of the chromatography was measured continuously by a mass detector using absorbance (AU) at 254 nm in (**A**) followed by a gamma-detector with amplifier (output in mV) in (**B**). The time lag between the occurrence of the mass peak from DHE at 5.318 min (**A**) and the radioactivity peak from [^{11}C]DHE at 5.495 min (**B**) is expected from the serial arrangement of the mass and radioactivity detectors. The overlap of peaks proves the chemical identity of DHE and [^{11}C]DHE.

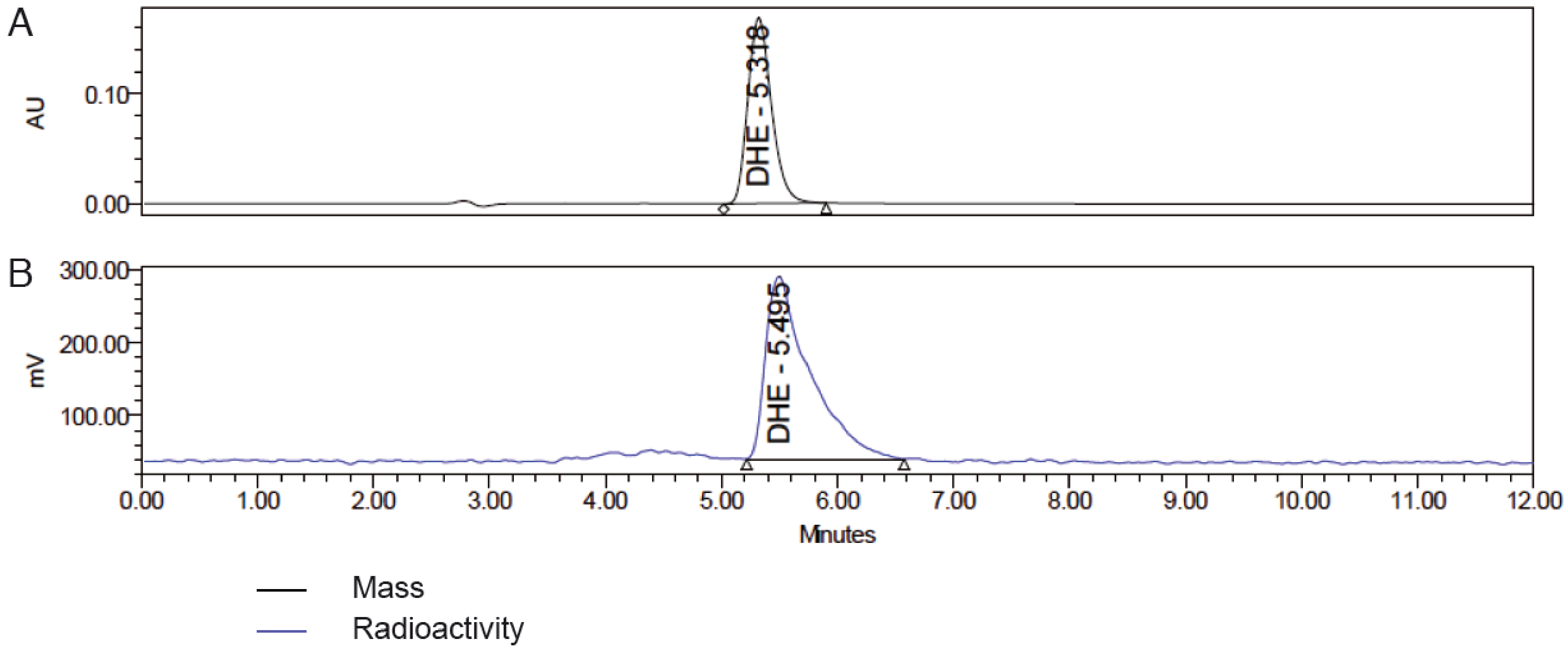
A
B
AU
0.10
0.00
DHE - 5.318
mV
300.00
200.00
100.00
DHE - 5.495
0.00
1.00
2.00
3.00
4.00
5.00
6.00
7.00
8.00
9.00
10.00
11.00
12.00
Minutes
Mass
Radioactivity
